# Packaging, Labeling, and Physical Characteristics and Sales Volume Assessment of Veterinary Antimicrobials in a Resource‐Limited Setting: Evidence From Hawassa Town, Ethiopia

**DOI:** 10.1155/vmi/5373047

**Published:** 2026-05-09

**Authors:** Lelisa Ache Yohannes, Yesuneh Tefera Mekasha, Melese Tilahun, Mikael Abreham, Gemmechu Hasen, Sileshi Belew

**Affiliations:** ^1^ Animal Disease Control and Clinical Coordinator, Liben District East Borena, Addis Ababa, Oromia, Ethiopia; ^2^ Clinical Trials Directorate, Armauer Hansen Research Institute (AHRI), Addis Ababa, Ethiopia, ahri.gov.et

**Keywords:** consumption, Ethiopia, Hawassa town, quality, sales volume, veterinary anthelmintic, veterinary antibiotic, visual inspection

## Abstract

**Background:**

Antibiotics and anthelmintics are widely used in veterinary medicine to prevent, control, and treat infectious diseases. However, in resource‐limited settings, the widespread circulation of poor‐quality medicines and their inappropriate use have become major obstacles to delivering effective animal healthcare. This study aimed to evaluate the quality of veterinary antibiotics and anthelmintics available across different levels of the supply chain. Additionally, it sought to estimate the sales volume and quantify the consumption of veterinary antimicrobials based on the population correction unit (PCU) in the pharmaceutical market of Hawassa Town.

**Methods:**

A facility‐based cross‐sectional study was conducted in Hawassa city. A total of 44 samples encompassing antibiotics (*n* = 22) and anthelmintics (*n* = 22) were collected from purposively selected veterinary drug retail outlets (*n* = 5) legally operating in Hawassa city. The samples were subjected to visual inspection using the World Health Organization (WHO) visual inspection checklist. In addition, the sales volume of antibiotic and anthelminthic medicines was assessed based on published peer‐reviewed journals and international guidelines such as the United States Food and Drug Administration, the WHO for animals (IOE), and the European surveillance of veterinary antimicrobials. Microsoft Excel and Microsoft Word were used to encode and analyze the whole quantitative data. Finally, the result is presented through tables, figures, and narrated text.

**Results:**

Out of 44 veterinary antimicrobials, 50% (11/22) of the samples did not comply with standard requirements, while all anthelmintic samples (*n* = 22) complied. All veterinary antimicrobials were found to be imported from foreign manufacturers. The average daily consumption of veterinary antibiotic and anthelmintic active pharmaceutical ingredients (APIs) in Hawassa city was found to be 4547 (60%) and 3073 (40%) grams, respectively. The total consumption of veterinary antibiotics adjusted for food‐producing animal biomass was 0.112 mg/kg. Tetracycline (51%) and albendazole (55.8%) were relatively the most consumed (sold) medicines.

**Conclusion:**

The study reveals significant quality concerns with veterinary antibiotics in Hawassa city, where 50% failed the WHO visual inspection, while anthelmintics fully complied. The high daily consumption of tetracycline and albendazole indicates a narrow reliance on a few drugs, raising concerns over antimicrobial resistance. The dependence on imports, mainly from China and India, indicates the need for stronger regulatory oversight and improved local capacity to ensure the safety and effectiveness of veterinary medicines.

## 1. Introduction

The provision of quality animal health service requires sustainable and adequate availability of safe, effective, and quality‐assured veterinary medicines as one of the critical elements [1]. However, the use of ineffective, poor‐quality, harmful medicines in animals has been recognized as one of the contributing factors influencing treatment, prevention, and control of both infectious and noninfectious animal diseases and resulting in therapeutic failure, exacerbation of disease, antimicrobial resistance development, and sometimes death [2]. It also undermines and diminishes the confidence of the public in animal health systems, animal health professionals, and pharmaceutical manufacturers and distributors. This points to the need for a stringent regulatory system for veterinary pharmaceuticals to ensure the quality, safety, and efficacy of veterinary medical products [3]. Such a system is also essential for promoting trade and supporting broader socioeconomic development.

According to the recent World Health Organization (WHO) report, 10% of all medicines circulating in low‐ and middle‐income countries are of poor quality [4]. In addition, a study conducted in Tanzania mainland revealed that the majority of the collected samples, 92% (219/238), failed to comply with product information requirements. The most observed deficiencies in product information were inadequate information on the package insert (94.1%, 224/238), inappropriate storage conditions (55.5%, 132/238), and the absence of a Tanzania registration number (27%, 64/238) [5]. The study from Cameroon showed that 69% of veterinary drugs sold did not meet international quality standards [6]. Similarly, a study conducted in 2001 across five regions of Cameroon assessed the quality of anthelmintics containing albendazole or levamisole, as well as trypanocidal drugs containing diminazene or ethidium (bromide or chloride). The results showed that 47% of the 34 samples obtained from both official and informal markets failed to meet international quality standards, highlighting widespread concerns about the quality assurance of veterinary medicines in the region [7]. The issue of substandard veterinary medicines extends beyond Cameroon. In Senegal, a study found that 67% of veterinary medicinal products did not meet regulatory quality standards. Notably, oxytetracycline‐based antibiotics exhibited the highest rate of nonconformity, with 93% of samples failing to comply with established specifications. These findings point to serious gaps in quality control and regulatory enforcement within the veterinary pharmaceutical sector [8]. Studies conducted in Benin, Togo, Mali, Mauritania, and Chad have revealed that veterinary medicines distributed through the parallel (unofficial) market are often of substandard quality. These findings underline the significant risks posed by unregulated pharmaceutical channels, which can undermine animal health, compromise food safety, and contribute to the development of antimicrobial resistance [9–11].

Globally, there is an increasing trend in the use of medicines in animals in both developed and developing countries to prevent and control animal diseases and to improve livestock production and productivity. Antibiotics and anthelmintics are among the most commonly used veterinary antimicrobial medicines employed for control, prevention, and treatment of infections caused by most disease‐causing pathogens [12]. However, such large‐scale use of veterinary medicine is mainly attributed to the global revolution in livestock, and aquaculture to satisfy the demand of a growing food population has contributed to the emergence of antimicrobial resistance [13, 14]. The problem of AMR is becoming beyond expectation, and globally, it is estimated that by 2050, the annual deaths of people attributed to antibiotic‐resistant infections will be roughly 10 million, and Africa alone will account for about 4.15 million [15]. There is evidence indicating an association between antimicrobial use and the development of AMR in human and veterinary medicine. Although increasing awareness has been raised in health facilities and communities [16], the extensive use of antimicrobials in the livestock sector remains a global concern; for example, in many countries, including Ethiopia, antimicrobial agents are widely available with virtually no restriction or controls over their use [17].

Monitoring of antimicrobial use is critical to understanding possible areas of risk for the development of resistance [18]. Therefore, the documentation of the antibiotic consumption both in humans and animals, as well as AMR, is an urgent global one‐health need [19]. Due to this, global action plans for integrated surveillance of AMR and AMU have been developed by the WHO in collaboration with the Food and Agriculture Organization (FAO) and the World Organization for Animal Health (OIE) to obtain accurate data on AMR and AMU across concerned sectors [20]. Data on antimicrobial consumption provide information on which antimicrobials are used in what quantities and allow for the assessment of trends over time at global, country, or health facility levels [21, 22].

However, countries with developed surveillance systems have little information on the appropriate use of antimicrobials in animals [23]. The problem is huge when it comes to countries like Ethiopia, where there is a poor culture of a quantitative data recording system. To overcome such problems, taking the experience of countries that used different modalities, such as sales data to estimate AMU, is important [24]. The AMU data can be derived from marketing authorization holders, wholesalers, or feed mills, and is relatively easy to obtain [25]. This study aimed to assess the regulatory compliance, packaging, labeling, and physical characteristics of veterinary antimicrobials; estimate the sales volume of antibiotics and anthelmintics; and quantify antimicrobial consumption per population correction unit (PCU) in Hawassa, Ethiopia, where data on medicine use and quality are limited despite widespread use of antibiotics and anthelminthics due to prevalent bacterial and parasitic infections.

### 1.1. Research Questions

The study heavily relies on three proposed research questions, including: (1) What is the quality status of packaging, labeling, and physical characteristics of veterinary antimicrobials available in the market of Hawassa city at various levels of the supply chain? (2) What is the magnitude of sales volumes of selected veterinary antimicrobial drugs available in the Hawassa city pharmaceutical market? (3) What is the quantity of the consumption of veterinary antimicrobials per PCU of the city?

By incorporating the research questions into a practical approach, the study provides the degree of compliance of veterinary drug outlets, and the sales volumes of selected antimicrobial classes of drugs were performed, providing baseline data for veterinary pharmaceutical policy maker, drug manufacturers, and academic researchers for future study needs.

## 2. Materials and Methods

### 2.1. Description of the Study Area

The study was carried out in Hawassa city. Hawassa is the capital of the Sidama regional state administration, and it is 275 km from Addis Ababa. It geographically lies between 4°27′and 8°30′ N latitude and 34°21′ and 39°1′ E longitude at an altitude of 1790 m above sea level. The area receives an average annual rainfall ranging from 800 to 1000 mm. The mean minimum and maximum temperatures of the area are 20.1°C and 30°C, respectively. The mean relative humidity is 51.8%. Hawassa city consists of eight subcity administrations, each with legislative, executive, and judicial organs [26]. In Hawassa city, an estimated 120,866 cattle, 51,405 goats, 49,377 sheep, and 1,224,503 poultry were found.

### 2.2. Study Design

A cross‐sectional, facility‐based study was conducted in the Sidama region, specifically targeting the main city of Hawassa. The study encompassed all subcities known to have a high concentration of veterinary drug shops. Data were collected from 11 veterinary drug outlets within each selected subcity to ensure comprehensive and representative findings.

### 2.3. Sampling Techniques and Data Collection Procedure

The study employed a purposive sampling technique to collect veterinary antibiotic and anthelmintic samples from legally operating drug outlets in Hawassa city. A total of 44 samples (*n* = 22 antibiotics and *n* = 22 anthelmintics) were collected from five retail veterinary drug shops that were selected based on their legal status and location within subcities known for high veterinary drug transactions. This nonrandom, targeted approach ensured that the study focused on formally recognized and operational outlets, which were most likely to reflect the official market practices. Data collection was carried out using structured checklists tailored for visual inspection developed from the WHO inspection tool [27], and veterinary market assessment was performed using data collection tools developed from peer‐reviewed published journals [27–29] and guidelines [28, 29]. The checklist was used to examine the quality attributes of medicines, including packaging, labeling, and other physical characteristics based on standardized inspection guidelines. Each collected product was coded and documented with detailed information such as the product name, manufacturer, active ingredients, dosage form, and strength.

In parallel, sales volume data were collected to estimate antimicrobial consumption. This involved gathering records from retail outlets and veterinary clinics over a 2‐week period, with data being updated every two to 3 days. The study adopted a retail audit method, wherein field visits were made to record the initial and final stock levels of each medicine type, adjusting for any additions or disposals. This method reduced reliance on recall and ensured higher accuracy in estimating actual sales. To complement these records, digital invoices submitted to government revenue offices were also analyzed, especially from outlets that had integrated digital reporting systems. Sales data were then converted into active pharmaceutical ingredient (API) weights using conversion standards from the OIE. Finally, the total antibiotic and anthelmintic consumption was expressed as milligrams per kilogram of livestock biomass, a standard metric known as the PCU, to facilitate a meaningful interpretation of usage relative to the food‐producing animal population.

A number of methods for measuring sales volumes have been identified, namely, reviewing providers’ sales records, asking providers to recall their sales volumes over a given period, conducting exit interviews with customers, and retail audits [30]. The retail was used to collect the sales data. Retail audits involve visiting a panel of outlets to collect stock information at regular intervals; at each visit, fieldworkers measure the stocks of an entire product category and ask the shopkeeper about any volumes added and/or disposed of during the visit interval. The volume of sales for each shop during the period is then estimated by subtracting the stock at the end of the period from the stock at the initial visit, corrected by any additions/disposals during the period [31]. The retail audit approach could be perceived to provide more accurate responses as it does not rely on respondents’ ability to remember their sales volumes. Therefore, the retail audit approach was used to precisely the data result. However, as it requires at least two visits to the same outlets within a given period (or a week), it is likely to be more costly and logistically complex than relying on records or provider recall (already reported/registered data from wholesalers) [31].

### 2.4. Statistical Calculation and Interpretation

The study used different statistical approaches to estimate the sales volumes of veterinary antimicrobials in the selected animal health settings. The volume of sales was expressed as the amount of antimicrobial substances in tons or kg and is usually completed by information on the substance and its pharmaceutical form [32, 33]. The total antibiotic and anthelmintic consumption of the city was calculated per month or day as PCU. In the antimicrobial calculation, there is an expression of dose strength by international unit, which is difficult to use as mg; therefore, the IU was converted to mg by using the IOE conversion standard [34]. The animal population per country or region is expressed as a PCU, which is related to the number of animals per animal species to the respective live weights. The calculated results in mg per PCUs differed widely between the countries or regions in nearly all antimicrobial classes, accounted for in part by different distributions of animal species. Countries with a high percentage of pigs and poultry seem to use more mg (antimicrobials) active ingredients per PCUs than others [35]. Fortunately, countries like Ethiopia do not consume or consume an insignificant amount of pork compared to other parts of the world [36, 37].

While several methodologies have been developed so far for the calculation of animal biomass by several surveillance groups, none could be directly used for the OIE global database [28]. These methodologies utilize available data on animal population detailed by production class, estimates of live animals’ weight, import/export data, number of animals used for breeding purposes, and total annual population of production groups living less than 1 year (i.e., poultry, veal calves, fattening pigs, lambs, and kids). At the global level, such detailed data are not yet available for many countries [34]. Hence, consumption of different antimicrobials has different patterns of utilization in different places or regions of the world.

The European surveillance of veterinary antimicrobial consumption and the United States Food and Drug Administration were utilized to determine the antimicrobial sales quantitatively to estimate the biomass of animals or the population at risk [38]. The definition of the population at risk of being treated is a crucial variable for which several different calculation approaches are available [39]. One concept is to identify the biomass, or live weight at risk of being treated by multiplying the number of produced or lived animals with the respective expected body weight at typical treatment age. The expected body weight differs between animal species, age, or production groups (Supporting File 1). Additionally, average animal weights at the typical age of treatment for European country standards used for the current study are also presented in Supporting File 2.

The key formulas (35) used for calculating daily defined doses for animals (DDDvet) using equation (1), and PCUs were calculated using equation (2):

(1) nDDDvet=total amount of active substance used mgdefined daily dose for animals DDDvet in mg,

where nDDDvet: number of daily doses for a particular antimicrobial, total amount of active substance used (mg): sum of API sold over the study period, and DDDvet (mg): standard daily dose of that antimicrobial for its main indication and route of administration in animals.

AMU per PCU (mg/PCU) was calculated using the following equation:

(2) AMU per PCUmgkg=total amount of antimicrobial used mg total animal biomass,

where AMU per PCU: antimicrobial usage normalized by animal biomass (PCU), and animal biomass (PCU): estimated by multiplying the number of animals per species by their average live body weight at treatment.

The study also formulated a statistical approach for slaughtered animals such as cattle, bovine, sheep, goat, and poultry to estimate the biomass. The data for the number of cattle slaughtered in the study area were acquired from the city administration’s abattoir. However, the cattle slaughtered outside the abattoir had no available data, so they were not included in the calculation:

(3) slaughtered cattle biomass kg=total number of cattle slaughtered×average live weight kg.

The bovine (all calves, bulls/heifers, and cows) biomass was calculated according to the following principles: Bovine biomass was calculated by multiplying the representative weight determined for each subregion by the census population of bovines for each country within the city:

(4) total bovine biomass=census population of bovines×mean live weight×P.Pop.calves+mean live weight−P.pop.12−rs+mean live weight×P.Pop.adults,

where P.pop calves = P.pop young 1−3 years, P.pop adults (above 3 years). The proportion (P.pop) of calves is young (between 1 and 2 years of age) and adults in the total living cattle population.

Slaughtered sheep and goats: 20 kg is the average number of slaughtered sheep and goats used in this calculation, consistent with the European standard:

(5) biomass kg=number slaughtered×average live weight kg.

The live sheep and goat were calculated as per the European standard calculation of the biomass of sheep and goats according to the following equation:

(6) live weight×number slaugthered+census population−numberslaughtered1.5×75 kg,

where (live weight × number slaughtered) represents the expected biomass of sheep and goats slaughtered in a country/specific area in 1 year, and (census population − (number slaughtered)/(1.5 )) × 75 kg represents the expected biomass of animals retained for breeding purposes, calculated with the following considerations: 1.5 is the average number of breeding cycles per year and the standard weight of a breeding small ruminant in Europe is 75 kg [40].

Therefore, the average body weight used in biomass calculation of sheep and goats < 1 year, 1–2 years, and above 2 years old were 18, 27, and 30 kg and 15, 25, and 30 kg, respectively, having considered the available literature about local. The poultry biomass (live + slaughtered) is calculated using the following equation:

(7) poultry biomass=total chicken population−2−week chicks×1 kg.

In this regard, a 1 kg of average live weight was applied for all mature poultry, in line with international standards. The youngest chicks were excluded from the calculation as they are not commonly treated or slaughtered.

### 2.5. Data Analysis

The sales volumes and visual quality inspection data obtained during the survey were summarized and appropriately organized. Microsoft Excel and Microsoft Word were used to encode and analyze the whole quantitative data acquired from the survey. Percentages and frequencies, together with tables, figures, and narrated texts, were utilized to present the scientific findings of the study.

### 2.6. Operational Definition

*Quality*: The quality of veterinary antimicrobials is determined based on their compliance with the WHO visual inspection checklist.

*Sales Volume*: The total quantity of each selected veterinary antibiotic and anthelmintic product dispensed by the study outlets during the data collection period.

*Compliance*: A veterinary antimicrobial product is classified as “compliant” if it fulfills all criteria specified in the WHO visual inspection checklist applied in this study.

## 3. Results

### 3.1. Visual Inspection‐Based Quality Assessment of Veterinary Antimicrobial Products

The visual inspection results revealed significant disparities in quality compliance between veterinary antibiotics and anthelmintics. Of the 44 total samples assessed, 50% (11/22) of the antibiotic products failed to meet WHO visual inspection standards, whereas all anthelmintic samples were fully compliant. This highlights a notable quality concern specific to antibiotics, suggesting potential lapses in manufacturing practices, labeling, or regulatory oversight. The most commonly violated parameter was the absence of API and dosage information (10/22 samples). Overall, 25% of the total samples (11/44) failed at least one visual inspection criterion, indicating widespread issues related to product traceability and identification, especially among antibiotic formulations. Detailed results are provided in Table 1.

**TABLE 1:** Visual inspection results of samples of veterinary antibiotics and anthelmintic medicines.

Medicines (*N* = 44)	Total nonconformities	Nonconformities with external packaging identifications				Nonconformities with traceability on external packaging	
		API name	Leaflet	API/dose	Exp. date	Name and address	Batch no
Antibiotic (*n* = 22)	11	7	6	10	8	9	8
Anthelmintic (*n* = 22)	0	0	0	0	0	0	0
Overall noncompliance	11	7	6	10	8	9	8
Overall percentage	25	15.9	13.6	22.7	18.1	20	18

### 3.2. Global Supply Chain of Veterinary Drugs: Country‐Wise Distribution in Hawassa Town

The study on the global supply chain of veterinary drugs in Hawassa Town revealed a notable dependency on imports, particularly from China. Among 22 samples of both antibiotics and anthelminthics assessed, the majority originated from China, with 13 samples in each category. India was the second most significant contributor, especially for anthelminthics, supplying samples (*n* = 9). This heavy reliance on Chinese and Indian manufacturers indicates the dominant role of Asian countries in the veterinary pharmaceutical market in the study area, while European countries such as Holland and Belgium contributed only to antibiotics, with no recorded supply of anthelminthic (Table 2).

**TABLE 2:** Country of origin of samples of medicines.

Manufacturer	Antibiotics	Anthelmintic
China	13	13
Holland	5	0
India	2	9
Belgium	2	0
Total	22	22

Interestingly, none of the antibiotic or anthelminthic products was sourced from Ethiopia, reflecting a complete dependence on foreign manufacturing for these essential veterinary drugs. This lack of local production raises concerns regarding the sustainability and resilience of the veterinary drug supply chain in the region, particularly in times of global disruptions.

**FIGURE 1:**
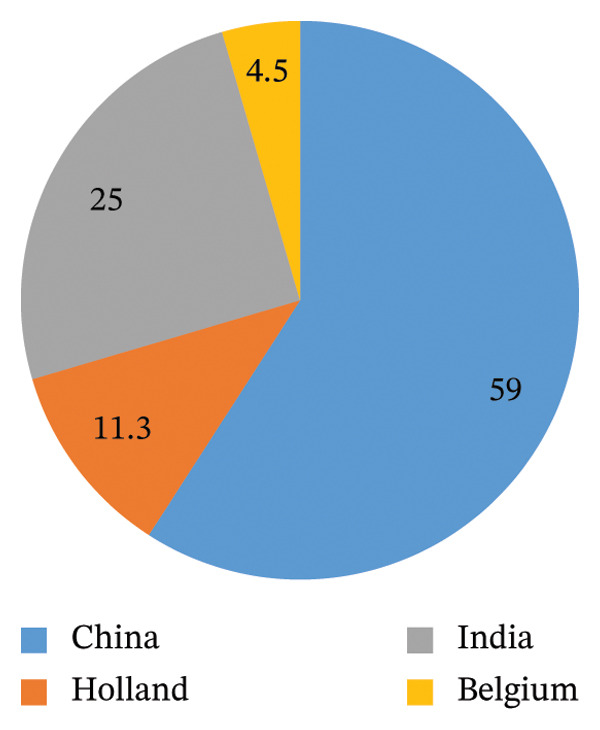
The percentage originality of the source of veterinary antibiotics and anthelminthics.

Furthermore, the majority of the veterinary drug samples analyzed 59% originated from China, followed by products from India, which accounted for 25% of the total samples, indicating it as the second‐largest source (Figure 1). These findings may reflect market accessibility, pricing, and regulatory factors that influence sourcing decisions in East African countries.

### 3.3. Sales Volume Assessment Results of Selected Antimicrobial Veterinary Drug

The study reveals that on average, 4547 and 3078 g of veterinary antibiotics and anthelmintics belonging to different classes were sold (consumed) per day, respectively. The sales volume assessment of selected anthelmintic veterinary drugs reveals a dominant reliance on oral administration, accounting for 99.6% of total usage, with injectable formulations making up only 0.35%. Among the drugs evaluated, albendazole, a benzimidazole‐class anthelmintic, represents the highest share at 55.8% of monthly usage, followed by oxyclozanide (17.07%) and tetramisole (11.64%). This heavy dependence on a few key oral drugs suggests their widespread availability, affordability, and ease of use in the field. Ivermectin was the only drug used both orally and by injection, yet its total contribution was relatively low (0.39%), which may reflect its high potency, targeted indications, or the logistical limitations of injectable formulations in rural settings. The detailed information on the monthly consumption (in grams) of various anthelmintics is presented in Table 3.

**TABLE 3:** Anthelmintic used per month based on route of administration in grams.

Anthelmintic class	Anthelmintic type	Orally used	% orally used	Injection	% injection	Oral + injection	% (oral + injection)
Salicylanilides	Oxyclozanide	13,696.75	17.13	0	0	13,696.75	17.07
Imidazothiazoles	Levamisole	2812.78	3.51	0	0	2812.78	3.5
Imidazothiazoles	Tetramisole	9343.65	11.68	0	0	9343.65	11.64
Benzimidazoles	Fenbendazole	4402.74	5.51	0	0	4402.74	5.57
Benzimidazoles	Albendazole	44,780.50	56	0	0	44,780.50	55.8
Microcyclic lactones	Ivermectin	33.36	0.042	281.5	100	314.86	0.39
Benzimidazoles	Triclabendazole	4886.85	6.11	0	0	4886.85	6.09
Total		79,956.65	100	281.5	100	80,238.13	100
Percentage		99.60%	—	0.35%	—	100%	—

From anatomical classification perspectives, oxytetracycline (*n* = 51%) (Table 4) and benzimidazole (67.28%) (Table 3) medicines were the most consumed antibiotics and anthelminthics, respectively, followed by sulfonamide (18.28%), beta‐lactam penicillin (*n* = 14.9%), and aminoglycoside (*n* = 14.85%), as can be presented in Table 4.

**TABLE 4:** Antibiotic used per month based on route of administration in grams.

Antibiotic class	Type	Oral (power)	Oral%	Injection	% injection	Externally applied	External%	Oral + injection + Ext.	% (oral + injection + ext)
Oxytetracycline	Oxytetra.Hc	36,125.50	81.88	24,192.10	32.68	30.47	100	60,348.07	51.11
Sulfonamide	Sulfadimidine	7992	18.12	13,589.33	18.33	0	0	21,581.33	18.28
Beta‐lactam/aminoglycoside	Pen‐strip	0	0	33,760	45.56	0	0	33,760	28.65
Fluoroquinolones	Enrofloxacin	0	0	80	0.11	0	0	80	0.068
Aminoglycoside	Gentamycin	0	0	570	0.77	0	0	570	0.48
Beta‐lactam	PPG (powder)	0	0	709.2	0.96	0	0	709.2	0.6
Macrolides	Tylosin	0	0	1170	1.59	0	0	1170	0.99
Total		44,117.50	100	73,910.63	100	30.47	100	118,058.61	100
Total percentage		37.3%	—	62.6%	—	0.025	—	100%	—
Possible animals consumed the antibiotics		Poultry alone		All (cattle, poultry, goats, &) sheep		All		All	
Antibiotics used DDD/PCU (mg/kg)		1.44		0.069		Trace		0.112	
% DDD/PCU		144		6.9		Trace		11.2	

The study also identified the sales volumes as per outlet levels. The outlets labeled as letters A, B, C, D, E, F, G, H, I, J, and K to prevent their being privileged. Accordingly, the outlet denoted by the letter “C” was the highest seller of antibiotics (*n* = 32%) and anthelmintics (*n* = 26.37%). No veterinary drug outlets were involved in the sales of veterinary drugs. For instance, as can be seen in Table 5, outlet K did not active in sales of anthelminthic (%) but actively engaged in the antibiotics pharmaceutical market (*n* = 0.25%) (Table 5).

**TABLE 5:** Sales volume of antibiotics and anthelminthic per outlet levels.

List of outlets	Antibiotics (%)	Anthelminthic (%)
A	18.16	10.25
B	16	7.69
C	32	26.37
D	2	2.10
E	1.4	6.02
F	4.3	23.68
G	4.68	16.45
H	0.84	2.88
I	5.3	2.03
J	15	1.88
K	0.25	—

### 3.4. Antibiotic Consumption Assessment Results in the Study Area

The study revealed that, among the major food‐producing animals exposed to the veterinary antibiotics and anthelmintics, cattle (*n* = 90.43%) accounted for the highest biomass (kg), followed by ovine (3.38%), caprine (3.26%), and poultry (3%). Despite that, poultry (84.67%) was the most numerous food‐producing animal, followed by cattle (8.36), caprine (3.55%), and ovine (3.41%) animals in the study area (Table 6). The survey also indicated that the total amount of antibiotics used adjusted for PCU of food‐producing animals was found to be 0.112 mg/kg (11.2%), while 1.44 mg/kg (144%) was found for oxytetracycline and sulfonamide antibiotics, which are supposed to have been consumed only by poultry in the oral route (Table 5). In addition, this study revealed that out of 4547 g of antibiotics consumed per day by all food‐producing animals in the study area, 37.35% of these antibiotics were consumed by poultry in the oral route . Furthermore, the finding showed that from orally consumed antibiotics by poultry, oxytetracycline and sulfonamide, respectively, representing 81.88% and 18.22%, made up the highest portion, respectively (Table 5).

**TABLE 6:** Animal population of the study area.

Species of animals	Poultry	Cattle	Goat	Sheep	Draft animal	Swine	Total number
Respective number of species	1,224,503	120,866	51,405	49,377	3836	200	1,450,187
% (All)	84.44	8.33	3.54	3.4	0.26	0.013	100
% Food producing	84.67	8.36	3.55	3.41			100

In this study, data for slaughtered animals were available only for bovines that were slaughtered in the abattoir from the Hawassa city administration abattoir (Supporting File 1). Despite the lack of the other slaughtered and animals’ category data, it was attempted to calculate these crucial data from the national average estimated animals slaughtered and animals’ category data, as well as in the referenced year, assuming that the study area animal population was evenly distributed as the national data (CSA, 2019/20). Consequently, the total PCU was calculated to be 40,615,099 kg for all food‐producing animals. The study area data acquired from the city’s livestock and fishery resource department revealed that poultry (84.67%) constituted the largest number followed by cattle (8.36%), goat (3.55%), and sheep (3.41) (Table 6). Swine and equines were not considered as food‐producing animals in Ethiopia and insignificant number that they contributed to the total biomass number too.

## 4. Discussion

Globally, the issue of quality of life has become a great deal. Thus, improving quality of life through compressive approach called “One Health,” which encompassed animal, human, and environmental health, is gaining attention and considered as the cornerstone in this regard. Hence, prudent use of quality, safe, effective medicines is critical in the provision of quality health services in both human and veterinary sectors. In the present study, the quality and use of veterinary medicines were assessed in Hawassa, which is one of the relatively large cities in Ethiopia. In the study, a total of 44 veterinary medicine surveys were performed. From this, veterinary antibiotics (*n* = 22) and anthelmintics (*n* = 22) were subjected to the WHO visual inspection tool. The study found that about 50% (11/22) of antibiotics did not comply with the assessment, while all the anthelmintics complied. This made the over nonconformities (25%, 11/44). Although only visual inspection or physicochemical analysis does not guarantee the exact quality of medicine, either of them tells us something snapshot about the quality of available drugs [41]. Nowadays, veterinary antibiotics are being consumed by animals more than by human beings. The utilization of antibiotics, coupled with indiscriminate use by animal owners, has made it the most used veterinary drugs among all other antimicrobials [42, 43]. However, these high demands and supply chains of producers and consumers may compromise the quality of antibiotics available in the market. In this regard, this may not tell us details about the quality; furthermore, veterinary drug quality surveys and physiochemical studies are required.

The study also indicates that 95.4% of veterinary antibiotics and anthelmintic drugs had brand/market name, which means of 44 veterinary medicines only two that were generic drugs. Utilizations of either generic or brand medicines are largely dependent on the socioeconomic and behavioral status of the consumer, but generally nongeneric (brand) medical products are more expensive than their counterparts’ generic products regarding the cost. In fact, generic drugs can cost 30%‐60% less than its brand counterpart [44]; even it can be as less cheap as 80% [45]. The trick is that since generic manufacturer does not have the research development, marketing, promotion, and other costs of the brand drug manufacturer, they are able to sell the drug at low cost [46]. Even though certain characteristics may differ such as probably color/name and nonactive ingredients, both the generic and brand medicines have the same purity, strength, and stability, and they also go through the same quality approval by FDA [44, 45]. Animal medicines are formulated in injection, oral bolus/powder, or externally applied formulations. Hence, most probably, there is no placebo effect on them as brand drugs do on human patients [47]. Therefore, the wide availability of cheap and quality generic drugs is preferred by animal owners to treat their animal patients. On the other hand, the wide availability of cheap drugs may be a source for inappropriate utilization of medicines as reported by [46]. With regard to the finding, however, the wide availability of brand expensive animal drugs may expose the farmers to the search for cheap illegal marginal/unofficial drugs, which may available near to their neighborhood. Unofficial markets are mostly unknown by governmental regulatory bodies, which finally exposes the animal owners to fake, substandard, and ineffective animal drugs [48]. Although there is not enough published literature on marginal illegal circulation of veterinary drugs in Ethiopia, ineffective veterinary drugs reported by several researchers [49–52] and this could be because of farmers for the search of cheap animal drugs, or because of low quality of the available drugs. Additionally, these fake substandard medicines may lead to antimicrobial resistance and other economic impacts on the farmers such as death of animals, cost for medicines, and time spent to cure the animal.

The study also revealed that almost all veterinary antibiotic and anthelmintic were manufactured by foreign pharmaceutical companies, such as china (60%), India (25%), Holland (11.3%), and Belgium (4.5%). These veterinary medicines are probably either imported from or produced there in the county by introduced foreign pharmaceutical countries. 70%–90% of medical products in developing countries are imported from developed European countries [53]. Medicines imported from developed countries or produced domestically may not guarantee the quality of the medicines. A study in Nigeria revealed this reality almost half of randomly selected antibiotics and antiparasitic drugs did not comply with the set standard, and in fact, these drugs were labeled as manufactured by Belgium, Holland, Switzerland, and the UK, as well as from less developed countries [48, 54]. In addition, AED medicine produced locally in Africa had better packaging than manufactured in China and India [55]; this may support local production of medicines.

Medicines can be manufactured by anybody, or any developing or developed country as long as they comply with good manufacturing practices (GMP). Remember, GMPs encompass several characteristics of the drugs such as standardized production of the API, proper labeling, and packaging materials that protect the APIs from external distorting factors, even appropriate transportation of the medicine after manufacturing [56–58]. However, common harmonization is a big problem and has not been solved yet in the pharmaceutical world and between developing and developed countries as well [59]. Despite the fact that quality‐assured medical products are the right to health of all individuals in the world, unfortunately, poor‐quality medicines and prevalence of diseases are common in the resource‐limited settings. Terribly, manufacturers and distributers selling their medical products in and for poorly regulated developing countries are not compelled by stringent regulatory and legal mechanisms to systematically implement GMP [54, 59–61]. On study conducted in Kenya, Gabon and Madagascar sampled from official and unofficial market supply chains indicated that overall 32.2% of the tablets were of poor quality. Furthermore, the prevalence of poor‐quality medicines were increased in samples supplied by public facilities and manufacturer located in China [55]. Here, we need to understand that the quality compromise may not always manufacturer, or somebody else, but a quality of medicine may be compromised on different chains such as improper production, transportation, storage, and utilization. Poor compliance with GMP standards due to accidental human mistakes/errors, such as insufficient resources, infrastructures, expertise, or other mistakes, can be tolerated and corrected over time humanly.

Apart from these, deliberately not giving enough attention to the lifesaving medicines exportable to less developed countries is an inhumane deed and a crime too [60, 61]. However, the reality present on the ground is that the quality of drugs for export from developed to developing countries is still determined through a much less rigorous evaluation than for the domestic market in Europe and other developed countries. Efficacy and safety of the drugs are not evaluated at all [54]. These substandard medical products manufactured in either developing or developed countries have huge effects on the health of humans and animals. The effects of these substandard drugs may not be clear at the time of treatment. In fact, they can be clinically effective, or ineffective, or unclear to the consumer at the time, and finally lead to drug resistance as well [48]. Ineffective veterinary medical products reported in different parts of the country could be because of substandard prevalence of poor‐quality medicines, drug resistance, and other factors. Nevertheless, domestically manufacturing medical products has more than economic implications and has benefits such as quality medicines for citizens, preparedness for disease outbreaks, and others such as job creations [53].

The study also showed that the physical characteristic assessments, such as shape, color, size, breaks/cracks, and contamination/foreign particles, had no nonconformities, or defects. Physical characteristics of medical products reveal something related to the quality of the drug and play a great role to identify the quality of the drugs in poorly equipped settings [62]. However, the visual surveys of medicines are only snapshot of the market status of the quality of the medicines. It does not mean that medicines that are nonconfirm in physical characteristics are good quality [5]. Physical contaminations of medicines are recurrent problems, especially in poorly developed countries and leading to fatal conditions. It was reported that a local donation of ringer’s lactated infusion was contaminated with a fungal growth and was discovered in first physical observation and with subsequent investigations revealed that weakness in bottling and quality control procedure of denoting countries [54]. In addition, physical characteristics such as cracks/breaks on capsules/bolus may indicate long storage conditions and poor transportations/managements of drugs. In addition, although it may not be used for quality assessments of veterinary medicines, color and size of a given brand may have great placebo effects on human patients [47].

The study also revealed that 45.4% (10/22), 31.8% (7/22), 27% (6/22), and 36.4% (8/22) of antibiotics had no identifications of API per dosage, API’s name, leaflet inserted, and expiry date explicitly on their external packaging, respectively. Moreover, 41% (9/22) and 36.4% (8/22) of all veterinary antibiotics’ external packaging material had not traceability information of name‐with‐address of manufacturer and batch number, respectively. GMP includes all good at producing API, packaging, labeling, and all quality assessments from production area until the drug reaches to the consumers [56–58]. Packaging may be defined as different components, which protect mechanically, or chemically the active pharmaceutical product from the time of production until it is used by the consumer, such as bottle, vial, closure, cap, ampoule, and blister [49]. Drug labeling means any written, pictorial, or other descriptive material that gives information about the veterinary drugs, including cartons, vials, and leaflets. All finished drug products available for market should be identified by labeling at their primary and secondary packaging, as required by the national legislation; the labels should not be easily erased and/or detached [63].

The label bears information and statements such as “for animal treatment only” or “for veterinary use only,” as well as the trade name, generic name, active ingredient, and other added substances; the withdrawal period; restrictions on use; batch number; storage instructions and handling precautions; directions for use (including dose rate, route, duration of treatment, and frequency of application); adverse effects; cautions; contraindications; manufacturing date; expiry date; net contents with a clear unit of measurement; and the name and address of the manufacturer or the company or person responsible for placing the product on the market [64]. The packaging of a pharmaceutical product is aimed at ensuring that veterinary medicines arrive safely in the hands of users. Packaging may be classified as primary or secondary. Primary container is in physical contact with the veterinary drug (e.g., bottle, blister pack, tube, syringe), whereas secondary packaging is the immediate packaging around the primary container (e.g., carton and leaflets/inserts) [65]. The choice of primary and secondary packaging materials depends on the degree of protection required, compatibility with the contents, the filling method and cost, and the convenience of the packaging for the user (e.g., size, weight, method of opening/reclosing, legibility of printing) [66, 67].

Although there are shortage of literature in Ethiopia, or specifically of the study area, unpacking or interchanging the secondary packaging materials is a common practice in many LMICs [55]. Veterinary medicines follow many channels and depend on many players in the supply chain before reaching the consumer. There are many points where quality can be compromised, and often, there are no adequate systems to monitor distribution [68]. In the present study, many veterinary drug sellers may not have understood the benefits of secondary packaging materials, and as a result, external packaging could be removed, leaflets might have been interchanged among drugs, or lost at some chains of the importation process; labeling information on the drug may also fade away because of long storage or other factors without the drugs get expired. However, unpacking or removing the secondary packaging materials exposes the API to adverse temperatures and humidity, which may compromise the quality of the drug by accelerating the deterioration and further oxidation and/or reduction of the API [39]. The problems of low stability of medicines API are doubled in hot and humid climates such as sub‐Saharan Africa [69]. A review reported by a guy indicates that half of the generic versions of Ramipril tablets were substandard after 3 months of storage under temperature‐stressed conditions [55]. This deterioration of pharmaceutical chemicals was accelerated when the secondary packaging materials were removed or replaced with low‐quality materials [55]. Additionally, veterinarians and animal owners may become confused when there is no labeling information on secondary packaging materials, which are often substituted with lower quality alternatives. Furthermore, the absence of proper secondary packaging may indicate poor drug quality, suggesting that the active ingredients have been exposed to adverse temperatures and humidity. Veterinarians, animal owners, and governmental bodies should discourage the use of veterinary medical products that lack complete or adequate packaging materials.

The study also discussed the sales volume and consumption trend of selected antimicrobial class of drugs. The study revealed oxytetracycline (51%), sulfonamide (18.2), beta‐lactam (14.9), and aminoglycosides (14.8) were the leading consumed antibiotic classes. They totally make up about 99% of antibiotic consumed in the study area, while the remaining 1% is made up by macrolides and fluoroquinolones. This finding agrees with a range of antibiotics commonly used in the area (Hawassa city) except last two class of antibiotics [51] class and also in Addis Ababa [50]. Similar findings in consumption of antibiotics were reported in German [70] and southwestern Nigeria [71], except that the percentage consumed, and that aminoglycosides (Germany) and sulfonamide (Nigeria) were replaced by other classes of antibiotics. Reasons for differences in percentage and variety of antibiotic classes consumed could be alternative availability of drugs, prevalence of bacterial diseases, prescription behavior, and up‐datedness of veterinarians, as well as the awareness of animal owners treating animals by themselves.

The result also revealed that benzimidazole (67%), salicylanilides (17%), and imidazothiazole (15%) were the most consumed anthelmintic groups; together, they constitute more than 99% of the anthelmintics used, while the remaining amount held by macrocyclic lactones (ivermectin). Similar anthelmintic classes were reported in Ethiopia [80]. In this study, benzimidazole alone holds 67% of anthelmintic consumed, of which albendazole makes up about 83% and 51% of the benzimidazole group and total anthelmintic consumed, respectively. Although there are differences in anthelmintic types, almost similar studies were reported, in terms of albendazole utilization, by university of Gondar veterinary clinic [72], eastern Ethiopia around Jijiga [73], and southern Ethiopia [74].

However, the result of this study is less than the result found in central Ethiopia anthelmintic prescribed or consumed [50]. The differences may arise from wide availability, and its consistent utilization may be its broad spectrum nature of the albendazole. The time/season of the year, disease epidemiology, prescription behavior of veterinarians, and animal owners’ knowledge may contribute to the variation in utilization of different anthelmintic. However, this consistently using of albendazole without alternating exposes the type of anthelmintic to be less and less effective against the worm and leads to so‐called antimicrobial resistance [75]. In different parts of the country, such a pattern of utilization of a single type of anthelmintic may be suggestive of one cause of anthelmintic resistance observed in animal [49, 52, 76].

The finding also revealed that oxytetracycline alone constitutes 51% of antibiotics consumed by food‐producing animals. Similar result was reported in the country [50–52]. This result is higher than consumed in the UK (40.5%) in 2014 [77] and also much greater than result reported [71] in southwestern Nigeria (33.6%), but less than the result reported in a longitudinal study in Tanzania (66%) [78]. Tetracycline is quite popular among many veterinarians and importers because of its broad spectrum of antibacterial activity and affordability [79]. Tetracycline is also widely and extensively used in treating diseases like anaplasmosis. It is also used in early stages of theileriosis and several supportive therapies in viral infection in animals like cattle [80]. Soluble tetracycline is also preferred as growth promoters, and these could be the reason for its most consumed antibiotic [78]. Additionally, availability of other alternative antibiotics, the policy shift of the country, or region, disease epidemiology, prescription pattern, and knowledge of animal owners could be the cause of differences in utilization. Places or countries having higher number of poultry and pigs consumed much more antibiotics than other having less number of these food animals. For instance, in the United Kingdom, poultry and pigs alone consumed around 61% of antibiotics in 2014 [77]. Furthermore, in Ethiopia, prescription or dispensing and utilization of antibiotics irrationally, or inappropriately are becoming common in animals [50, 51], and public settings [81, 82] too. All these factors collectively will rush the failure of treatments and development of antimicrobial resistance in both settings.

In the study, 4547 g (60%) and 3073 (40%) grams of antibiotics and anthelmintic were consumed by the animals per day. Furthermore, this result revealed that antibiotic consumed is 1.5 times more than the anthelmintic consumed per day. This result is nearly in line with the prescribed antibiotics (54.5%) and anthelmintic (38.9%), in which antibiotic consumed was 1.4 times more than anthelmintic consumed reported in central Ethiopia veterinary clinics [50]. However, the report by [41] showed that 27% of the antibiotics were prescribed irrationally for treating viral disease secondary complication with parasite cases. Although not exactly known for this result, this may be suggestive of irrational use of antimicrobials all over the country in veterinary as well as public health settings [51, 81, 82].

The total sales/consumption of veterinary antibiotic adjusted for food‐producing animal population was 0.112 mg/PCU (11.2%). Although it was impossible to draw up the consumption of antibiotic for each species of food‐producing animals, it showed that 0.6 mg/PCU calculated for poultry alone. This literally indicates that 112 μg of any antibiotic active ingredient consumed was for 1 kg of biomass of all food‐producing animals considered in the study area and 600 μg active ingredient for 1 kg of study area poultry population. This result may not exactly indicate the sales of all veterinary antibiotics consumed in the study area and could not be merely compared with countries, or specific regions that have developed antibiotic surveillance system [83]. However, this finding may be considered as a baseline data for antimicrobial consumption for the study area and considering the animal population change over time with antimicrobial consumption is crucial aspect of surveillance of antimicrobial consumption. There are several reasons by which these data may vary from the exact antimicrobial consumption of the study area. The time/season of the year, under/over reporting of sales drugs, the time length of data recordings, biomass composition of the region/area, the wide availability of specific antibiotic, and other factors may be source of variations. To continue, however, the surveillance of the antibiotic consumption is important to discover the pattern of antibiotic utilizations, resistance development of specific antibiotic, and policy devising for animal sectors related to antibiotic consumption and the growth of animal biomass.

### 4.1. Limitation of the Study

The amount of antibiotics and anthelmintic reported in this study may not exactly reflect the actual consumption of antimicrobials in the study area. Rather, there may have been over/underreporting of sales volume by veterinary drug dispensers, refusal of some dispensers to give data/records, illegal veterinary drug circulations, and shortage of time to collect year‐round data.

Since veterinary drugs pass through several supply chains until they reach the consumers (animals), external packaging, and leaflets might have been interchanged among drugs or lost altogether; labeling information on the drug can also fade away because of long storage or other factors without expiring, so these may give false interpretations of the drugs in the market.

## 5. Conclusion and Future Direction

This study indicates significant concerns regarding the quality and utilization of veterinary antimicrobial products in Hawassa Town, Ethiopia. Visual inspection revealed that 50% of veterinary antibiotic samples failed to meet WHO standards, in contrast to full compliance among anthelmintic samples. Furthermore, the analysis of sales and consumption data demonstrated a disproportionately high usage of a few drug classes, most notably tetracycline and albendazole, raising the risk of antimicrobial resistance. The complete reliance on imported medicines, predominantly from Asian countries, further emphasizes systemic vulnerabilities related to quality control, traceability, and supply chain resilience. Future interventions should prioritize strengthening postmarket surveillance systems and enforcing strict regulatory standards to ensure the quality of veterinary pharmaceuticals. Building national capacity for routine physicochemical analysis, establishing a traceable drug distribution system, and enhancing the labeling and packaging of antimicrobial products are critical steps. Given the over‐reliance on a narrow spectrum of antimicrobials, efforts should be made to promote rational drug use through antimicrobial stewardship programs, updated treatment guidelines, and educational campaigns targeting veterinarians and animal owners. In addition, the establishment of a national antimicrobial consumption surveillance system using standardized metrics such as the PCU is recommended to support data‐driven policy decisions. Additionally, encouraging local manufacturing of veterinary drugs under GMP could reduce dependency on imports and improve quality assurance. Finally, fostering multisectoral collaboration between regulatory authorities, academia, veterinary professionals, and community stakeholders is essential to combat substandard drugs, safeguard animal health, and mitigate the growing threat of antimicrobial resistance.

## Author Contributions

All authors contributed equally to this manuscript.

## Funding

The authors have nothing to report.

## Ethics Statement

The study involving human participants was reviewed and approved by the Ethics Committee of Jimma University College of Agriculture and Veterinary Medicine. All patients/participants provided written informed consent to participate in this study.

## Conflicts of Interest

The authors declare no conflicts of interest.

## Supporting Information

Additional supporting information can be found online in the Supporting Information section.

## Supporting information


**Supporting Information 1** Supporting information 1: WHO visual Inspection checklist.

**Supporting Information 2** Supporting information 2: Ages and average estimated biomass of food‐producing animals.

**Supporting Information 3** Supporting information 3: Average animal weights at typical age of treatment for European countries standard.

## Data Availability

The original data generated were found within the article. Additional supportive information was attached as supporting.
