# Supplementary material for: Packaging, Labeling, and Physical Characteristics and Sales Volume Assessment of Veterinary Antimicrobials in a Resource‐Limited Setting: Evidence From Hawassa Town, Ethiopia
Source: Vet Med Int. 2026 May 9;2026:5373047. doi: 10.1155/vmi/5373047 (PMC13157313; doi:10.1155/vmi/5373047)
Supplement: Supplementary file 1 — Supporting Information 1 Supporting information 1: WHO visual Inspection checklist. [file VMI-2026-5373047-s003.docx]

# Supplementary File 1: WHO visual Inspection check list

**VISUAL INSPECTION CHECKLIST**

Name/identification of the product

Date: ___/____/_______

**A. PACKAGING YES NO OBSERVATION**

1. Is there an external packaging?

2. Is the external packaging intact?

3. Is the internal packaging intact?

4. Does the internal packaging provide-?

Clear information on the storage condition of the medicine?

**B. IDENTIFICATION YES NO OBSERVATION**

**B1.Does the external packaging carry the following information on the outer side**

5. Name of the active ingredient

6. The amount of active ingredient per dosage unit or packaging?

7. The expiry date in an uncoded form (i.e., Exp. Date 07/20, may20)?

B2.Does the internal packaging carry the following information on the outside

8. Name of the active ingredient?

9. The amount of active ingredient per dosage or packaging?

10. The expiry date in an uncoded form (i.e., Exp. Date 07/20, may20)?

**C.TRACEABILITY YES NO OBSERVATION**

**C1.Does the external packaging carry the following information on the outside:**

11. The name and address of the manufacturer or the company?

(Person responsible for placing the product on the market)

12. The batch number?

**C2.Does the internal packaging carry the following information on the outside:**

13. The name and address of the manufacturer or the company?

(Person responsible for placing the product on the market)

14. The batch number?

**D. PHYSICAL APPEARANCE YES NO OBSERVATION**

**D1.Powder for suspension, solution, and syrups for oral**

15. Is the color of the powder/solution homogenous?

16. Is it homogenous, free from lumps, clots, and foreign particles?

17. Are there clear instruction for preparing the solution/suspension-

and the quantity of liquid to be used?

**D2.Capsule/blister/tablet**

18. Have the capsule/tablet have the same shape, dimension/size, color, marks?

19. Are the capsules/tablets free from cracks, erosion, stains, and foreign particles?

**D3.Sterile liquids, powders for injection**

20. is the color of the internal container intact and airtight?

21. Is the color of liquid /powder homogenous?

22. Is the texture homogenous, free from lumps/clots, and foreign particles?
