# Supplementary material for: Packaging, Labeling, and Physical Characteristics and Sales Volume Assessment of Veterinary Antimicrobials in a Resource‐Limited Setting: Evidence From Hawassa Town, Ethiopia
Source: Vet Med Int. 2026 May 9;2026:5373047. doi: 10.1155/vmi/5373047 (PMC13157313; doi:10.1155/vmi/5373047)
Supplement: Supplementary file 2 — Supporting Information 2 Supporting information 2: Ages and average estimated biomass of food‐producing animals. [file VMI-2026-5373047-s002.docx]

**Supplementary File 2:** Ages and average estimated biomass of food producing animals

| **Animal species** | **Average estimated weight(kg)** | **Age** | **Biomass calculated** | **Respective biomass percentage** | **Species Total biomass kg** | **Biomass species %** |
| --- | --- | --- | --- | --- | --- | --- |
| Bovine | 92 | <1year | 1,997,228kg | 4.91 | 36,731,959 | 90.43 |
| Bovine | 226 | 1-3 years old | 4,799,336kg | 11.81 |  |  |
| Bovine | 315 | 3+ years old | 24,541,965kg | 60.42 |  |  |
| Bovine/slaughtered | 315 | 3+ years old | 5,393,430kg | 13.27 |  |  |
| Sheep | 18 | <1 year | 294,642 kg | 0.72 | 1,375,964 | 3.38 |
| Sheep | 27 | 1-2 year | 191,052 kg | 0.47 |  |  |
| Sheep | 30 | 2+years | 777,990 kg | 1.91 |  |  |
| slaughtered sheep | 20 | Assumption | 112,280 kg | 0.27 |  |  |
| Goat | 15 | <1 year | 246,060 kg | 0.6 | 1,327,490 | 3.26 |
| Goat | 25 | 1-2 years | 200,100 kg | 0.49 |  |  |
| Goat | 30 | 2+years | 810,090 kg | 1.99 |  |  |
| Slaughtered goat | 20 | Assumption | 71,240 kg | 0.17 |  |  |
| poultry/chicken | 1 | 12+ weeks all | 857,152 kg | 2.11 | 1,179,686 | 3 |
| Slaughtered poultry | 1 | Assumption | 322,534 kg | 0.79 |  |  |
| Total | |  | 40,615,099 kg | 100% | 40,615,099kg | 100% |
|  |  |  | **40,615 ton** |  | **40,615 ton** |  |
