# Supplementary material for: Packaging, Labeling, and Physical Characteristics and Sales Volume Assessment of Veterinary Antimicrobials in a Resource‐Limited Setting: Evidence From Hawassa Town, Ethiopia
Source: Vet Med Int. 2026 May 9;2026:5373047. doi: 10.1155/vmi/5373047 (PMC13157313; doi:10.1155/vmi/5373047)
Supplement: Supplementary file 3 — Supporting Information 3 Supporting information 3: Average animal weights at typical age of treatment for European countries standard. [file VMI-2026-5373047-s001.docx]

Supplementary File 3: Average animal weights at typical age of treatment for European countries standard

| **Animal category** | **Weight (kg)** |
| --- | --- |
| Slaughtered cows,bulls,or bullocks; dairy cows | 425 |
| Slaughtered heifers | 200 |
| Slaughtered calves, and young cattle; feeding cattle | 140 |
| Living sows | 240 |
| Slaughtered pigs | 65 |
| Fattening pigs | 25 |
| Living sheep | 70 |
| Slaughtered sheep and goat; fattening sheep and goat | 20 |
| Turkey | 6.5 |
| Broilers | 1 |
| Horses | 400 |
| Rabbits | 1.4 |
| Fish | Biomass slaughtered weight |
